# Spectrum of adverse cutaneous eruptions to nevirapine – a cross sectional study

**DOI:** 10.1186/1471-2334-12-S1-O9

**Published:** 2012-05-04

**Authors:** Leelavathy Budamakuntla, Prabhakar Basappa, DM Basavarajaiah, N Vidyashankar, C Madhura, N Shilpa, P Namitha, C Lalitha

**Affiliations:** 1 Bowring and Lady Curzon Hospital, Bangalore Medical College & Research Institute, Bangalore, India

## Background

HIV infection increases the risk of adverse drug eruptions. The reason for this is unclear; the mechanism probably involves drug specific cytotoxic lymphocytes. The aim of the study was to observe the spectrum of adverse cutaneous eruptions to nevirapine.

## Methods

PLHIV initiated on nevirapine based ART regimens between 2010 and 2011, were included in the cross sectional study. These hospitalized patients were followed up for the sequence of events. A detailed concomitant drug history was taken to rule out other impending drugs which can cause similar drug reactions.

## Results

In this analysis, 80% were females, 70% heterosexuals, 20% homosexuals and 10% intravenous drug users. Average age of patients was 31.70± 9.89 years. The spectrum of drug eruptions ranged from erythematous maculopapular generalized rash in 20% of patients to grade four SJS & TEN in 80%. Regression and inverse growth model was employed to find out the significant duration between the onset of nevirapine rash and initiation of ART. Mean duration of onset of nevirapine rash was 12.40±3.78 days, with mean CD4 count at the time of drug reaction was 162.10±32.61 microns/dl. It was observed that the correlation co-efficient (r=0.981) between the onset of nevirapine rash and CD4 count was significant (P≤0.05), which clearly depicts the association of the onset of nevirapine rash to low CD4 count (<200 microns/dL).

## Conclusion

NVP rash can show polymorphic manifestations, and the majority of them occurs within first 4-6 weeks of initiation of ART and is often associated with low baseline CD4 count.

